# The functional impact of nuclear reorganization in cellular senescence

**DOI:** 10.1093/bfgp/elab012

**Published:** 2021-03-23

**Authors:** Azucena Rocha, Audrey Dalgarno, Nicola Neretti

**Keywords:** cellular senescence, nuclear organization, chromatin, epigenome, aging

## Abstract

Cellular senescence is the irreversible cell cycle arrest in response to DNA damage. Because senescent cells accumulate with age and contribute to chronic inflammation, they are promising therapeutic targets for healthspan extension. The senescent phenotype can vary depending on cell type and on the specific insults that induce senescence. This variability is also reflected in the extensive remodeling of the genome organization within the nucleus of senescent cells. Here, we give an overview of the nuclear changes that occur in different forms of senescence, including changes to chromatin state and composition and to the three-dimensional organization of the genome, as well as alterations to the nuclear envelope and to the accessibility of repetitive genomic regions. Many of these changes are shared across all forms of senescence, implicating nuclear organization as a fundamental driver of the senescent state and of how senescent cells interact with the surrounding tissue.

## Introduction

Cellular senescence is the irreversible arrest of cell proliferation in response to stressors that cause irreparable DNA damage. Stressors such as telomere shortening, irradiation, oncogenic or oxidative stress, and exposure to genotoxic agents, trigger cell cycle arrest via the DNA damage response (DDR) signaling pathway [1].

Cellular senescence occurs at different life stages and has both beneficial and detrimental effects. During embryonic development, programmed senescence orchestrates tissue growth and patterning [2]. Senescence also plays a role in cellular plasticity and stemness [3] and facilitates tissue remodeling and healing [4]. In later life, the accumulation of senescent cells contributes to a wide range of age-associated diseases by promoting chronic inflammation [5, 6] and tumor progression [7]. The causal link between senescent cell accumulation and tissue dysfunction has been demonstrated in rodents, as clearance of senescent cells led to a significant increase in both healthspan and lifespan [8].

In this review article, we focus on the changes in nuclear organization in senescent cells and on their functional consequences, particularly on their roles in triggering an irreversible cell cycle arrest and the subsequent inflammatory response. Most studies have been conducted in commonly used models of senescence, including replicative senescence (RS) in response to telomere erosion through consecutive cell divisions [9], oncogene-induced senescence (OIS) caused by replication fork collapse due to the activation of oncogenes [10] and DNA-damage induced senescence caused by different DNA damaging chemical agents or increased levels of reactive oxygen species (ROS) [11, 12]. These forms of primary senescence can induce secondary senescence on neighboring cells through the release of inflammatory factors or via the NOTCH signaling pathway (NOTCH-induced senescence, or NIS) [13].

## The changing chromatin landscape

Chromatin changes significantly with senescence and these changes have important functional consequences. Post-translational modifications of histones such as methylation and acetylation create two distinct chromatin states: compact, inaccessible heterochromatin and open, accessible euchromatin [14]. Senescent cells display both a global loss and focal gains of heterochromatin [15–17]. Most prominently in OIS, senescence features the formation of senescence-associated heterochromatic foci (SAHF) [18]. All forms of senescence display broad changes in the landscape of histone variants and post-translational modifications [19–27]. These changes yield altered transcriptional programs, contributing to the senescent phenotype [24–27].

### Global loss and focal gains of heterochromatin

Early work on senescence supported the heterochromatin loss model of aging, which posits that senescence-associated changes in gene expression are due to the progressive loss of heterochromatin and the consequent transcription of otherwise-silenced genes [28]. In support of this model, drug-induced demethylation impairing heterochromatin formation shortens proliferative lifespan [29], and histone deacetylase inhibitors also induce premature senescence [30].

Heterochromatin is associated with H3K9, H3K27 and H4K20 methylation, low acetylation levels, and the presence of heterochromatin protein 1 (HP1) [14]. The loss of heterochromatin contributes to senescence as knocking down HP1α or SUV39H1, which methylates H3K9, induces premature senescence [31]. Recently, DGCR8 and ZKSCAN3 were found to stabilize heterochromatin. Deficiency in either protein causes premature senescence, and overexpressing either of the two diminishes the senescent phenotype. Both DGCR8 and ZKSCAN3 interact with heterochromatin components, including HP1 proteins [32, 33].

**Figure 1:**
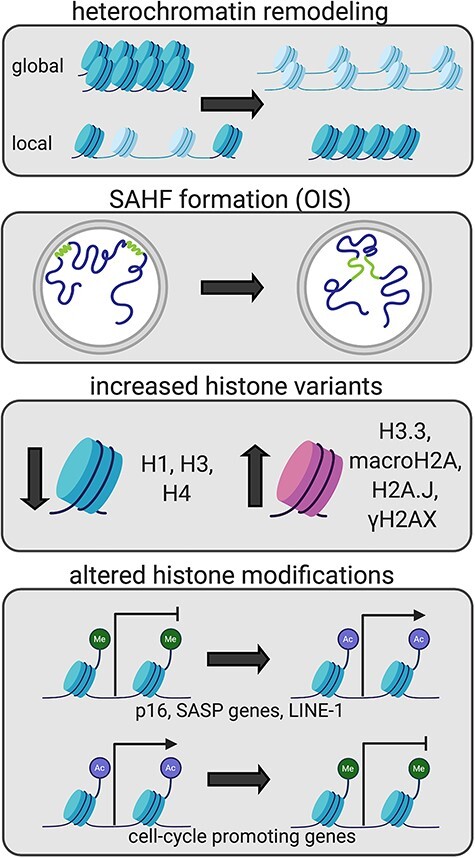
Senescence-associated chromatin changes. Globally, heterochromatin (shown in blue) is lost, but there are additional focal gains of heterochromatin [15–17]. In OIS, SAHFs (shown in green) are formed [18]. Canonical histones become less prevalent, whereas increased proportions of histone variants are observed [19–23, 47, 49]. Modifications to histones drive a senescence-associated transcriptional program [24–27, 65]. Acetylation = Ac, Methylation = Me.

Global loss of heterochromatin is contrasted with local gains in heterochromatin (Figure 1). In RS, although the loss of methylation occurs in gene-poor late-replicating regions associated with the nuclear envelope (NE), the gains of hypermethylation occur in promoter regions, including those of genes which, when repressed, inhibit cell cycle progression. The hypomethylation is attributed to aberrant localization of DNA methyltransferase 1 (DNMT1) [15]. Studies on chromatin accessibility support these findings, as RS cells show increased accessibility in gene-poor heterochromatin and decreased accessibility at promoters and enhancers [16]. Overall, chromatin accessibility increases in both OIS and NIS, including at gene-distal sites such as enhancers and repeat regions, although largely at separate sites in the two different types of senescence. NIS also shows a decrease in accessibility at gene-distal sites [17].

### Senescence-associated heterochromatic foci

SAHF are regions of compacted heterochromatin observed primarily in OIS (Figure 1). SAHF exhibit enrichment in H3K9me3, HP1 proteins, H4K20me3 and H3K27me3 around the SAHF periphery [18, 34, 35], all markers of heterochromatin. SAHF contribute to cell cycle arrest by forming on loci containing genes responsive to E2F, a transcription factor associated with cell cycle progression [18].

High-mobility group (HMG) proteins such as HMGA1 and HMGA2 have been implicated in SAHF formation [36]. HMG proteins are abundant non-histone regulatory proteins that associate with chromatin and alter its structure. HMGA, HMGB and HMGN are three distinct families of HMG proteins and differ based on their DNA binding motifs [37]. Ectopic overexpression of HMGA2 can induce senescence and SAHF formation. GSK3β, a Wnt pathway regulator, is essential for HMGA2-induced SAHF formation [38]. The Wnt pathway is associated with stem cell regulation, implicated in cancer and repressed in senescence [39, 40]. Additionally, NOTCH-signaling represses HMGA1, disrupting SAHF formation [17]. Another HMG protein, HMGB2, does not affect SAHF formation but promotes the senescence-associated secretory phenotype (SASP) [41], i.e. the secretion of soluble signaling factors, insoluble proteins, extracellular matrix components and proteases. The SASP creates a proinflammatory environment, which can lead to chronic inflammation, drive neighboring cells to senescence and aid tumor progression [42]. In OIS, HMGB2 localizes to SASP genes and prevents their incorporation into SAHF by fending off the spread of heterochromatin marks [41]. This contrasts with findings that indicate HMGB2 does not play a similar role in RS. Although there are *de novo* HMGB2 peaks observed with RS, these do not include loci associated with the SASP [43]. One plausible reason for this discrepancy is the heterogeneity of the SASP across different types of cellular senescence [13, 42]. Notably, OIS has a SASP profile distinct from other types of senescence such as RS. The OIS SASP features higher secretion levels of more general SASP factors such as IL-6 and IL-8 as well as the secretion of OIS-specific SASP factors including ENA-78 and G-CSF [42]. Additionally, NIS has a distinct SASP profile with prominent TGF-β [44]. Secondary senescence has a composite SASP profile with contributions from both paracrine and NOTCH signaling [45].

Finally, recent work has implicated nuclear pores in the formation of SAHF, as the increase in nuclear pores density observed in OIS is required for SAHF formation. The association of the nucleoporin translocated promoter region to the nuclear pore complex was shown to be necessary for SASP activation in OIS [46].

### Histone modifications and variants

The prevalence of canonical histone proteins decreases with senescence (Figure 1). In RS, stress from telomere shortening causes reduced expression of histones H3 and H4. The decreased quantity of histones compromises the chromatin landscape and thus amplifies local damage to a larger scale [21]. Levels of H1, a linker histone, also decrease in senescent cells containing SAHF [20].

Histone variants become more abundant as cells progress into the senescent state (Figure 1). For example, increased levels of macroH2A, a family of transcription-silencing histone variants, have been found in SAHF [19]. In OIS, macroH2A1, a member of the macroH2A family, redistributes with help from ATM, notably moving away from SASP genes and allowing their transcription [47]. ATM also mediates the DDR associated with senescence and phosphorylates the histone variant H2AX [48, 49]. In its phosphorylated state H2AX (γH2AX) may ‘anchor’ the ends of double-stranded breaks (DSBs) in close proximity, enabling repair [50]. Another histone variant, H2A.J, accumulates with DNA-damage-associated senescence. This rare variant is important to upregulating inflammatory and immune response genes, including those associated with the SASP [23]. Furthermore, the histone variant H3.3 becomes more prevalent in senescence, and its cleavage leads to cell cycle arrest through the silencing of cell cycle regulators [22].

Histone modifications also play significant roles in senescence by altering the transcriptional landscape (Figure 1). For example, H3K9ac and H4K16ac at the promoters of the SASP genes IL-8 and IL-6 increase with senescence, promoting their transcription. Sirtuin 1 (SIRT1), an NAD+-dependent protein deacetylase, normally prevents acetylation of these SASP genes, but its expression is decreased with senescence [24]. Additionally, The MYST-family histone acetyltransferase MOZ maintains H3K9ac and H3K27ac at several INK4A-ARF pathway inhibitors, including CDC6, EZH2 and E2F2, thus inhibiting senescence [25]. Accordingly, inhibiting MOZ promotes senescence [51]. p16 (p16^INK4A^) is an essential tumor suppressor and senescence-marker encoded at the INK4A-ARF locus. p16 inhibits cyclin d-dependent kinases CDK4 and CDK6, activating the G1-S cell cycle checkpoint and preventing proliferation [52]. The downregulation of EZH2, the aforementioned INK4A-ARF inhibitor, leads to DDR activation and thus senescence. Later, EZH2 downregulation is followed by H3K27me3 loss, notably at p16 and activation of the SASP [26]. Additionally, in RS, histone acetyltransferase p300 drives a senescence-associated transcriptional program because of its induction of super-enhancers enriched in several acetylation marks and H3K4me1 [27].

## Alterations to the NE

A key function of the NE is to protect the genetic material enclosed in the nucleus. Insults to the NE result in pathological states because of genomic instability and altered gene regulation. RS and OIS cells display NE blebbing and the formation of cytoplasmic chromatin fragments (CCFs). Nuclear blebbing can also be observed in Hutchinson–Gilford progeria syndrome (HGPS) cells and in aged cells from healthy individuals because of progerin accumulation. HGPS is a rare disease characterized by premature aging because of mutations in LMNA (lamin A/C) causing progerin, a protein product with an internal deletion [53]. Nuclear lamins are intermediate filament proteins that play a role in maintaining the structural properties of the nucleus, as well as in the regulation of DNA replication, transcription and chromatin organization [54].

Alterations to NE integrity have profound functional consequences in senescent cells. The release of DNA fragments from the cell nucleus into the cytosol triggers an innate immune response via the recognition of cytosolic DNA by cGAS (cGAMP synthase). cGAS activates STING, inducing the phosphorylation and nuclear translocation of IFN (interferon) regulatory factors (IRFs) and promoting the SASP [55–60].

CCFs contain genomic DNA, the DNA damage marker γH2AX, but not the DSB repair regulator 53BP1, and heterochromatin markers H3K9me3 and H3K27me3 [61–63]. This suggests that CCFs are derived from damaged heterochromatic regions and involve the DDR. Whether the content of CCFs preferentially contains specific chromatin elements remains unclear. CCFs are later degraded by an autophagic/lysosomal pathway [61]. The autophagy protein LC3 binds directly to lamin B1. This interaction mediates lamin B1 degradation upon oncogenic insults, playing a key role in reinforcing cellular senescence [62]. The lamin B1 receptor (LBR) is also lost when DNA is damaged by ɣ-radiation in cancer cells, which causes changes in chromatin structure including blebbing, micronuclei, and CCFs and promotes senescence in cancer cells [64]. The mechanisms that initiate CCF formation in the nucleus are not well understood, and whether CCFs preferentially contain specific genomic elements is still not clear. Evidence exists that a large fraction of these elements originates from LINE-1 retrotransposons [65].

Mitochondria have been shown to play a role in CCF formation via mitochondria-to-nucleus retrograde signaling via ROS and the stress-activated c-Jun N-terminal kinase (JNK). Senescent cells lacking mitochondria show a strong suppression of CCFs [66]. Additionally, 53BP1 has been shown to interact with JNK and negatively regulate CCF formation.

Lukášová *et al*. also reported that euchromatin and heterochromatin are extruded from the nucleus independently. In irradiated MCF7 cells, most DNA-carrying vesicles contained low-density chromatin, lamin B1 and lamin A/C, but lacked LBR and heterochromatin markers, which is indicative of euchromatin release. Senescent cells, on the other hand, extruded CCFs through ruptures in the lamin A/C meshwork. The CCFs were made of heterochromatin not coated by lamins but sometimes attached to LBR. Similarly, several other NE proteins are downregulated in OIS [67].

NE rupture frequency correlates inversely with lamin A/C levels and can be reduced in genome-edited LMNA knockout cells by the inhibition of actomyosin contractility or the acetyl-transferase protein NAT10 [68]. Also, the downregulation of lamin B1 increases CCF levels, whereas the overexpression of lamin B1 impairs CCF generation [61, 62]. Given both lamin A and lamin B are altered in aging, it is possible that NE ruptures also increase with aging. lamin A depletion leads to the weakening of the NE, where cytoskeletal pressure might originate NE blebs, culminating in rupture [69].

Extracellular vesicles (EVs) contain exosome components. DNA damage activates the ceramide synthetic pathway, leading to increased senescence-associated EV biogenesis [70]. EVs contain proteins, lipids and chromosomal DNA fragments, which indicates that exosome secretion may play a role in removing harmful cytoplasmic DNA from cells. The inhibition of exosome secretion results in the accumulation of nuclear DNA in the cytoplasm, leading to ROS-dependent DDR and a senescence-like cell cycle arrest in human cells [71]. Jeppesen *et al*. investigated micronuclei and found their morphology and markers were consistent with their identity as multivesicular endosomes, proposing a new model for active secretion of extracellular DNA through an autophagy- and multivesicular-endosome-dependent but exosome-independent mechanism [72].

## Chromosome reorganization

Recent technological advancements have revolutionized our knowledge of chromosome organization. Hi-C, a chromosome conformation capture technique, has revealed the genome is organized hierarchically in three-dimensional (3D) space into open and active A compartments and more closed and repressed B compartments [73]. Locally, chromosomes are organized into topologically associating domains (TADs), ~1 Mb regions with elevated levels of intra-domain contacts [74]. At higher resolution, DNA is folded into loops, formed through a process mediated by cohesin called loop-extrusion. DNA loops often connect promoters and enhancers and are associated with gene activation; convergent CCCTC-binding factor (CTCF) motifs are normally found at loop anchors [75].

One of the first studies on chromosome organization in an age-related context examined HGPS [76]. McCord *et al*. [76] performed Hi-C on HGPS fibroblasts and identified a loss of compartments in late passage HGPS cells, albeit the compartment loss, the study still identified an increase in compartment switching (i.e. switching from A to B or B to A) as compared with controls. Compartment switching can affect gene expression. Regions that switch compartments tend to reflect the transcriptional level of the compartment they join [77].

Relatively low-resolution Hi-C analysis of OIS cells informed how chromosome organization changes in senescence. The analysis indicated a sequence- and lamin-specific heterochromatic loss of local interactions; these regions were inaccessible, GC poor, enriched for H3K9me3 and associated with lamins. Although these regions lose local interactions, they also come together in space, which is indicative of SAHF formation [78]. This study contrasted their findings with those in HGPS to support a two-step formation of SAHF, as OIS and HGPS share similar changes in local interactions, but HGPS does not exhibit a gain of distal interactions. TAD boundaries were mainly conserved between proliferating and OIS cells.

**Figure 2:**
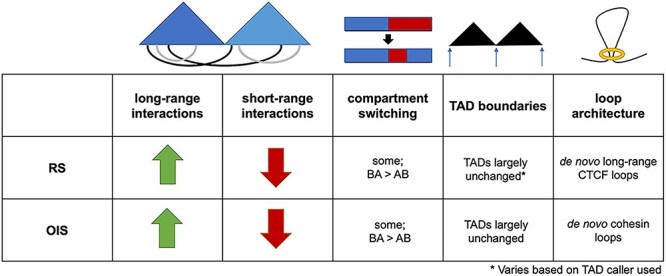
Overview of chromosome organization changes in RS and OIS. Long-range interactions increase while short-range interactions decrease [83]. There is evidence of compartment switching with more B to A than A to B compartment switches [82]. TADs are largely unchanged in both types of senescence, although a less commonly used method identified changes such as fusing and shifting TADs in RS [43, 79, 86]. In RS, there is evidence of *de novo* long-range CTCF loops formed across sites occupied by HMGB2 in proliferating but not senescent cells [43]. In OIS, *de novo* loops connecting enhancers and promoters were observed [86].

The first Hi-C study of RS found a decrease in long-range and an increase in short-range contacts. TAD boundaries were overall conserved between proliferating, quiescent and senescent cells, but a subset of TADs switched compartment in senescent cells with respect to both quiescent and proliferating cells (Figure 2) [79]. The study also reported a significant reduction in chromosome volumes and interpreted it as a consequence of their detachment from the lamina caused by the depletion of lamin B1 [80], which had been proposed previously as a potential mechanism for retrotransposon activation in senescent cells [16]. In contrast, Zirkel *et al*. investigated chromosome organization in RS using three cell lines at higher resolution and observed an increase in long-range interactions and only limited compartment switching. This discrepancy could be explained by this latter study using cells entering senescence (‘early’ senescence), whereas the former one used cells kept in a senescent stage for a long period of time (‘deep’ senescence). There was shifting, fusing and separating of TADs with RS [43]. However, these TADs were identified using a less commonly used method, which was not included in a study that evaluated and compared many TAD callers [81]. HMGB2 was identified as being depleted in the nucleus during senescence and was found to affect genomic architecture. HMGB2 is located at a subset of TAD boundaries, helps insulate CTCF loops, and its depletion is sufficient to form senescence-induced CTCF clusters, a reorganization of CTCF that occurs with senescence. In accordance with HMGB2’s insulating role, *de novo* long-range CTCF loops were observed with RS across locations where HMGB2 was formerly present in proliferating cells (Figure 2) [43].

More recent studies have examined both RS and OIS at higher Hi-C resolution [82, 83]. Sati *et al*. found a loss of local interactions and gain of distal interactions for both types of senescence (Figure 2). For OIS, compartmentalization increases because of the loss of A-B contacts and gain of B-B contacts. For RS, compartmentalization decreases because of the gain of A-B contacts and loss of A-A contacts [83]. Some compartment switching occurs, with a higher proportion of B to A switches than A to B switches (Figure 2). The regions undergoing compartment switching are significantly conserved between OIS and RS and exhibit transcriptional changes: upregulation in B to A switches and downregulation in A to B switches. Condensin is important to maintaining senescence, as it enforces the A compartment and is implicated in B to A transitions, allowing the expression of senescence-related genes [82]. Both RS and OIS feature senescence-associated heterochromatin domains (SAHDs), areas enriched for H3K9me3, which develop into SAHF in OIS. DNMT1 is important for SAHF formation because it increases the expression of HMGA2 [83]. The formation of SAHF alters gene expression. Although Iwasaki *et al*. found genes 500–700 kb from SAHF exhibit statistically significant downregulation, Sati *et al*. found that SAHF bring together specific loci to enable their gene expression; these include genes relating to cancer and inflammatory response, but do not include SASP genes. Polymer modeling revealed lamina detachment and SAHD decompaction may cause SAHF formation [83].

Cancer and senescence exhibit similar epigenetic changes. With colorectal cancer, the A and B compartments lose spatial segregation, becoming more homogeneous in 3D space. Additionally, there is a novel self-interacting compartment, I, intermediate to A and B, with distinct intermediate patterns of contacts between the A and B compartments within nuclear space. In tumors, the I compartment becomes hypomethylated, resembling the B compartment and enriched for H3K27me3. Very similar changes were observed with late passage fibroblasts, indicating that the compartmental changes are a feature of excess cell division rather than cancer and thus aid in impeding malignant progression [84].

Polymer modeling was able to recapitulate senescence-associated changes in chromosome organization. A polymer model with varying heterochromatin-heterochromatin and heterochromatin-nuclear lamina interactions identified four chromatin states, including those resembling growing cells, senescent cells and progeroid cells. According to the model, the transition between proliferating and senescent cells is abrupt and is stabilized by hysteresis [85].

Although previous studies mainly addressed senescence-associated changes in macro-domains such as A/B compartments and TADs, a recent study from the Narita lab focused on how chromatin loops change in OIS. OIS features altered enhancer-promoter interactions, indicative of loop formation, notably at the IL1 cluster, which contains important SASP and cell cycle-associated genes (Figure 2). OIS-associated changes in enhancer-promoter contacts support that inflammation-related genes are upregulated, and cell cycle-related genes are downregulated [86]. Some of these changes can be attributed to transcription-dependent cohesin repositioning following the observation of ‘cohesin islands’ by Busslinger *et al*. Cohesin islands form at the 3′ ends of activated genes after cohesin is loaded on to the transcription start site and progresses because of transcription, but there is inefficient offloading and no impeding CTCF; this creates *de novo* cohesin peaks and plausibly *de novo* loops [86, 87]. Regarding macro-domains, Olan *et al*. found TAD borders and A/B compartments to be mostly conserved with OIS, but the TAD containing HMGA2 was among those most changed (Figure 2) [86].

## Repetitive regions

Repetitive DNA sequences make up a major proportion of nuclear DNA in the eukaryotic genome and are composed of hundreds of thousands of repeated sequence motifs [88]. Repetitive DNA sequences act as nucleation centers for heterochromatin formation and are usually transcriptionally repressed. They are tandemly arrayed in the centromeric region of chromosomes, at telomeres at the end of chromosomes, or interspersed across the genome, such as transposable elements (TEs) [89]. These regions experience changes in organization during senescence.

### Telomeres

Telomeres are heterochromatic DNA repeat regions found at the ends of chromosomes that form protective structures for chromosome integrity. Telomeres gradually shorten with cellular replication, leading to a permanent cell cycle arrest known as RS [90].

Telomeres are redistributed to the nuclear center in late passage human fibroblasts [91], whereas in OIS cells they are preferentially associated with the nuclear lamina (NL) [92]. In mice, the loss of lamin A results in telomere accumulation at the nuclear periphery, in addition to telomere shortening, defects in telomeric chromatin and increased genomic instability [93]. This indicates that telomere relocalization might be associated with telomere dysfunction.

Telomeres are protected by t-loops, whose formation requires the shelterin component TRF2. lamin A/C reduction or progerin expression associated with premature aging disorders results in reduced t-loop formation and telomere loss, demonstrating the impact of the interaction between TRF2 and lamin A/C on chromosome structure [94]. In addition to telomere compartmentalization, lamins also affect telomere mobility in the nucleus [95].

The depletion of AKTIP, a shelterin-interacting protein, correlates with senescence-associated markers and recapitulates the progeroid phenotype in cells. AKTIP is required for replication of telomeric DNA, localizes at the nuclear periphery, interacts with A- and B-type lamins and affects lamin A expression in interphase cells. These results confirm AKTIP’s role in lamin-related processes and its effect on nuclear architecture, telomere homeostasis and cellular senescence [96].

Higher-order chromatin organization at telomeres has been reported to alter gene expression in cells, a phenomenon known as telomere position effect over long distances, suggesting a potential mechanism for the contribution of telomere shortening to aging. Also, Hi-C experiments on human chromosome 6p revealed that as telomeres shorten during rounds of cell division, the loss of gene-telomere interactions leads to alterations in the expression of genes near telomeres [97].

Telomeres contribute to the integrity of eukaryotic genomes by acting as sensors of both intrinsic and extrinsic stress. Additionally, telomeres aid in DSB repair. Uncapped telomeres lead to a double-strand DNA repair response, inducing the cells to become senescent [98]. Mammalian telomeres are protected from the DDR by the shelterin protein complex; removal of TRF2 from shelterin leads to the derepression of the DNA repair pathways, including the phosphorylation and accumulation of 53BP1 [99]. Dysfunctional telomeres and DSBs are more mobile than undamaged chromatin, and new studies have shown that 53BP1-dependent mobility of dysfunctional telomeres is a LINC/microtubule-dependent process that promotes non-homologous end-joining. However, this mechanism promotes the mobility of ionizing radiation-induced DSBs and contributes to their misrepair in poly ADP ribose polymerase inhibitor-treated BRCA1-deficient cells [100]. This feature of the DDR might lead to aberrant DNA repair in the presence of extensive damage.

DNA damage foci co-localize with telomere regions and increase in cardiomyocytes with age independently of telomere length, telomerase activity or DNA replication [101]. Recent studies induced regulated DSBs within telomeric DNA (T-DSB), indicating the DDR is more muted in response to telomeric DSBs. The muted response allows mitosis to proceed in cells with residual damage or fused chromosomes, leading to an increased number of micronuclei compared with controls. Additionally, after the induction of T-DSBs, there is increased phosphorylation of cytosolic DNA-mediated immune signaling pathway markers such as STING, TBK1 and IRF3. DNA sensor cGAS recruitment to CCFs correlates positively with the activation of immune signaling in response to telomeric DSBs, and this process triggered senescence in cells, independent of telomere length. This study confirms that imperfect DDR signaling because of dysfunctional telomeres, such as in cells with fused chromosomes, can cause the accumulation of chromatin fragments in the cytosol, leading to a premature senescence phenotype independently of telomere shortening [102].

### Centromeres

Centromeres are heterochromatic chromatin domains that display dramatic structural alterations in senescence. Histone variant CENP-A protein levels are diminished with age in human islet cells and are also reduced in senescent human primary fibroblasts [103, 104]. Additionally, the reduction of CENP-A by shRNA causes premature senescence in fibroblasts.

Human centromeres harbor a class of DNA repeats named satellites, which are normally constitutively repressed, but in RS cells, pericentric satellites HSATII distend and become accessible [16]. Additional studies confirmed the distension of centromeres, or senescence-associated distension of satellites, on pericentric satellite II and centromeric alpha satellite as an early event in both RS and OIS [79, 105]. The satellite sequences are also hypomethylated, consistent with distension and derepression [15].

Pericentric heterochromatin silencing at centromeres is crucial for genomic stability and protection against mitotic defects and senescence. SIRT6, a histone deacetylase, maintains the silencing of pericentric satellites by removing H3K18 acetylation in proliferative cells. The depletion of SIRT6 leads to senescence and accumulation of pericentric satellite transcripts, which in turn increases mitotic errors, chromosome missegregation and aneuploidy, cytoplasmic micronuclei, and cellular senescence [106]. Because SIRT6 is one of the first factors recruited at the sites of DSBs, it is possible that in the presence of extensive irreparable DNA damage, SIRT6 sequestration away from pericentric satellite DNA initiates centromere unraveling and distension.

Using chromosome-orientation fluorescent *in situ* hybridization and super-resolution microscopy to monitor centromeric repeat stability in human cells, Giunta *et al*. showed that the depletion of CENP-A and members of the constitutive centromere-associated network (CCAN) proteins leads to an increase in centromere aberrations. This suggests a role for CENP-A and CCAN in protecting centromere integrity. Additionally, CENP-A protects α-satellite repeat integrity [107]. The analysis of nuclei labeled with a CENP-B antibody and DamID to investigate the changes in genome–NL interactions in an OIS model showed that centromeres in OIS cells tend to move toward the nuclear lamina [92].

Anchoring of heterochromatin to the NE contributes to the spatial organization of chromatin structure in the nucleus. In cancer cells, heterochromatin is tethered to the inner nuclear membrane (INM) by LBR. Both LBR and lamin B1 are downregulated at the onset of cell senescence [108]. This downregulation leads to the detachment of centromeric repetitive sequences from the INM, relocation to the nucleoplasm and satellite distension, which in turn results in changes in chromatin architecture and gene expression [64]. Genotoxic stress conditions trigger transcriptional activation of centromeric repeats, followed by disorganization of centromeres with delocalization of nucleosomal CENP-A, which in turn leads to the accumulation of micronuclei [109]. The increase of satellite RNA has been shown to have effects also on human cancers. Genomic instability induced by satellite RNAs occurs through interactions with BRCA1-associated protein networks, which are required to stabilize DNA replication forks. Consequently, de-stabilized replication forks might promote the formation of RNA–DNA hybrids [110].

**Figure 3:**
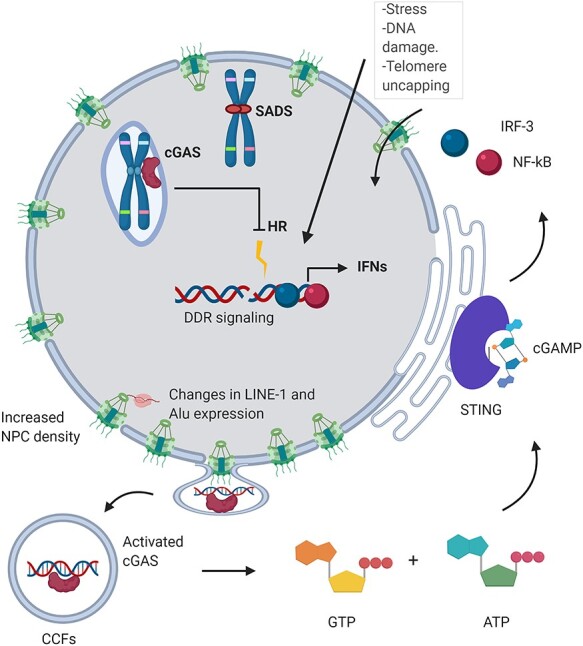
The DDR response is triggered as a response to stress (irradiation, ROS), DNA damage or telomere uncapping, leading to the senescent state. Changes of nuclear localization of telomeres disrupt their maintenance and homeostasis. Nuclear cGAS accumulates at LINE-1 s and centromeres [123], and it interferes with DNA repair [124, 125]. SIRT-6 sequestration to DNA damage sites impacts LINE-1 expression [120]. These changes lead to nuclear blebbing and the expulsion of CFFs [53], which elicit an interferon response via the cGAS-STING pathway, leading to chronic inflammation [58].

### Transposons

TEs comprise almost half of our genomes, and they are enriched in constitutive heterochromatin [111]. Retrotransposons are a type of TE that can mobilize themselves, integrating into the genome using a copy-and-paste mechanism. Interspersed elements, a type of retrotransposon, can be classified based on their length as short interspersed elements (SINEs) and long interspersed elements (LINEs). Alu elements are a type of SINEs that show upregulated transcription in senescence, and they are associated with persistent DNA damage foci and loss of efficient DNA repair in pericentric chromatin [112]. Within the LINE class, LINE-1 comprises about 17% of the human genome and is normally repressed [88]. LINE-1 expression can result in insertional mutagenesis, genomic rearrangements, deletions and DSBs [113–115]. Transposition can contribute to genetic disease, aging and cancer [116]. TEs also regulate gene expression by reshaping chromatin structure or by providing transcription factor binding sites [117, 118].

Cellular senescence is characterized by extensive epigenetic remodeling involving changes to the chromatin of Alu, SINE-VNTR-Alus and LINE-1 elements, which become more open. This derepression increases RNA expression and mobilization of these elements, stimulating the cGAS-STING pathway and eliciting a type-1 IFN response and the SASP [16, 65, 79]. Retrotransposons are repressed in heterochromatin by epigenetic factors, including DNMT1, SUV39h, HP1 and SIRT6. In senescent cells, LINE-1s are derepressed by the loss of epigenetic inactive marks and are activated by the transcription factor FOXA1 [65].

Retrotransposons are also activated in cancer cells as well as in old and progeroid mice [119–121]. SIRT6-deficient cells and tissues accumulate cytoplasmic LINE-1 cDNA. This triggers a strong type-I IFN response via activation of cGAS. Inhibiting LINE-1 replication extends the healthspan and the lifespan of SIRT6 KO mice [120]. SIRT7 has also been shown to play a role in the epigenetic transcriptional repression of LINE-1 in mouse and human cells. The depletion of SIRT7 leads to increased LINE-1 expression and retrotransposition by promoting interaction with lamin A/C and via H3K18 deacetylation, which is associated with gene repression [122].

Nuclear cGAS has been reported to localize preferentially to centromeres and LINE-1 elements, which points at an additional contribution of cGAS as a ‘transposition sensor’ [123]. Nuclear cGAS also interferes with homologous recombination repair in the nucleus, but the mechanisms by which this happens are yet to be elucidated [124, 125]. One hypothesis is that cGAS prevents strand invasion by Rad 51 by compacting chromatin via phase separation [126, 127] (Figure 3).

In mouse embryo fibroblasts, transfection with LINE-1 expression constructs induces an IFN-β response, dependent on LINE-1’s ORF2 endonuclease activity, suggesting that IFN-β induction requires active LINE-1 transposition. Additionally, either induced IFN-β or exogenous IFN-β inhibited LINE-1 transposition showed that IFN-β induced by LINE-1 transposition suppresses LINE-1 transposition in a negative feedback loop [128].

Recent studies have shown differential methylation of retrotransposons in chronic lymphocytic leukemia that modulates the expression of proximal genes [129]. LINE-1 hypomethylation is observed with increasing age and as a result of exposure to ionizing radiation *in vivo* [130, 131] and global genome hypomethylation can be observed during premature cell senescence induced by oxidative stress. Additionally, hydrogen peroxide treatment causes translocation from non-CpG-rich to CpG-rich areas of proteins such as DNMT1, DNMT3B and SIRT1 [132]. More studies are needed to better understand whether the general decrease of methylation observed in senescence preferentially occurs for a particular subset of TEs.

**Key Points**

- Changes to the chromatin landscape promote a senescence-associated expression profile including activation (p16, the SASP, LINE-1) and repression (cell cycle promoting genes).
- RS and OIS cells display NE blebbing and the formation of CCFs, which triggers an immune response via the cGAS-STING pathway.
- Genomic architecture is altered in RS and OIS, enabling the senescent phenotype through changes such as compartment switching and altered enhancer-promoter contacts.
- Repetitive regions of the genome, including telomeres, centromeres and retrotransposons, experience changes in organization during senescence.

## Notes

**Azucena Rocha** is a PhD candidate in the Molecular Biology, Cell Biology and Biochemistry program at Brown University. Her research focuses on the mechanisms of DNA damage sensing in senescence induction and aging.

**Audrey Dalgarno** is a PhD student in the Molecular Biology, Cell Biology and Biochemistry program at Brown University studying the connection between genomic architecture and function in the context of aging.

**Nicola Neretti**, PhD, is an Associate Professor in the Department of Molecular Biology, Cell Biology and Biochemistry at Brown University. His research focuses on transcriptional and epigenetic changes in cellular senescence and aging.
